# Supplementary material for: An Optimized Peptide Antagonist of CXCR4 Limits Survival of BCR–ABL1-Transformed Cells in Philadelphia-Chromosome-Positive B-Cell Acute Lymphoblastic Leukemia
Source: Int J Mol Sci. 2024 Jul 30;25(15):8306. doi: 10.3390/ijms25158306 (PMC11312813; doi:10.3390/ijms25158306)
Supplement: Supplementary file 1 [file ijms-25-08306-s001.zip › ijms-3037785-supplementary.pdf]

Supplementary Figure S1

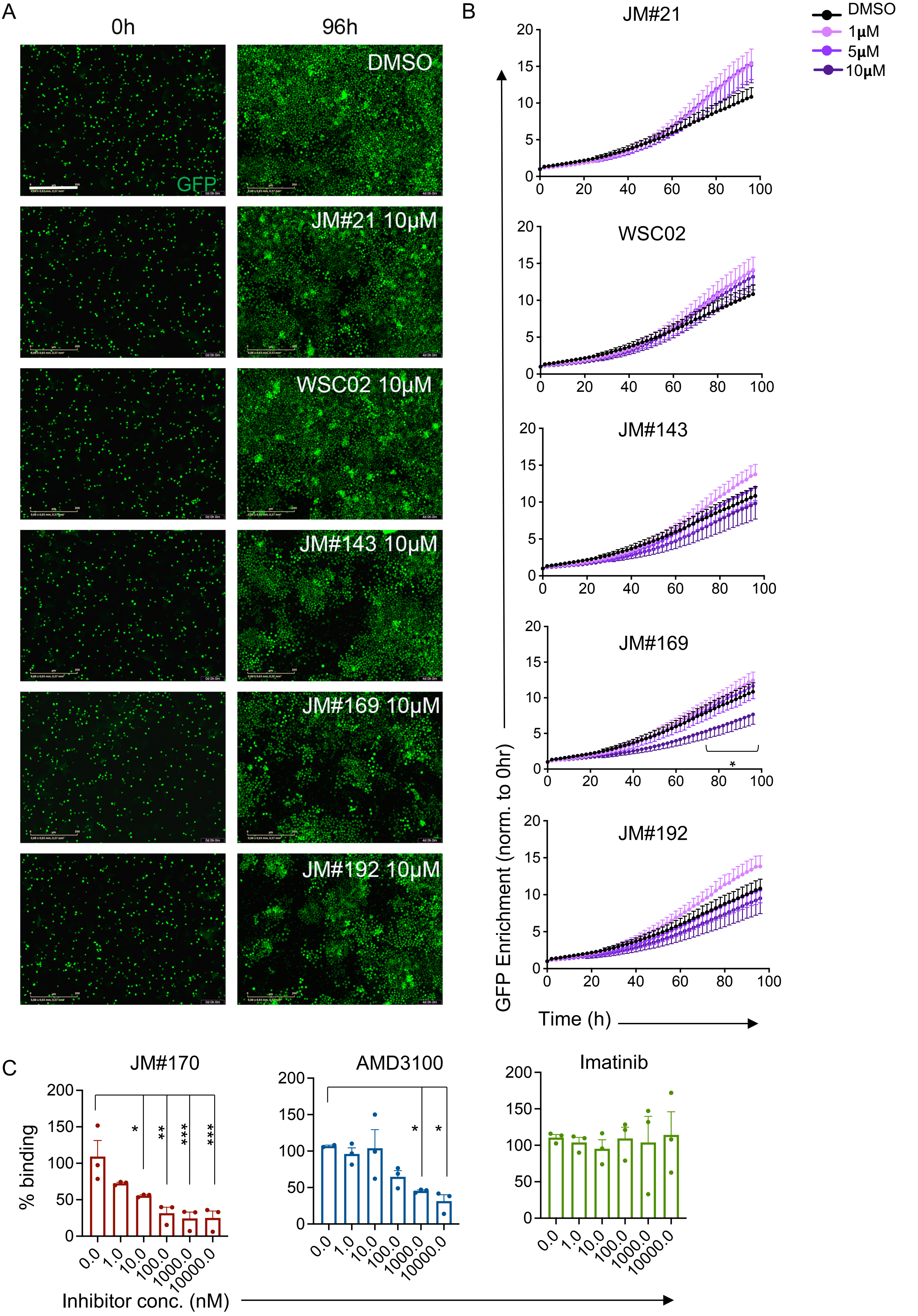

Effect of optimized EPI-X4 derivatives on cellular growth in BCR-ABL1 transformed mouse B cells. A. Real-time imaging of BCR-ABL1 transformed mouse B cells treated with solvent DMSO or 10µM of JM#21, WSC02, JM#143, JM#169 and JM#192 over 96 hours. Images are representative of 4-5 independent experiments. Scale bar- 200µm. B. Quantification of GFP enrichment as a marker of BCR-ABL1 cell growth in control DMSO and different (1, 5 and 10µM) concentrations of EPI-X4 derivative treated cells over 96 hours. The count of GFP^+ cells for each time point for each treatment is normalized with respect to the corresponding count at 0 hour. Graph represents mean ± SEM, n=4-5. Statistical analysis- two-way ANOVA with Dunnett's multiple comparison test. *p<0.05, **p<0.01, ***p<0.001. C. Anti-CXCR4 antibody (clone# L276F12B) competition assay in BCR-ABL1 transformed mouse B cells in presence of indicated concentrations of JM#170 (left panel), AMD3100 (middle panel) and Imatinib (right panel). Bar represents mean ± SEM, n=3. Statistical analysis- one-way ANOVA with Dunnett's multiple comparison test. *p<0.05, **p<0.01, ***p<0.001.

Supplementary Figure S2

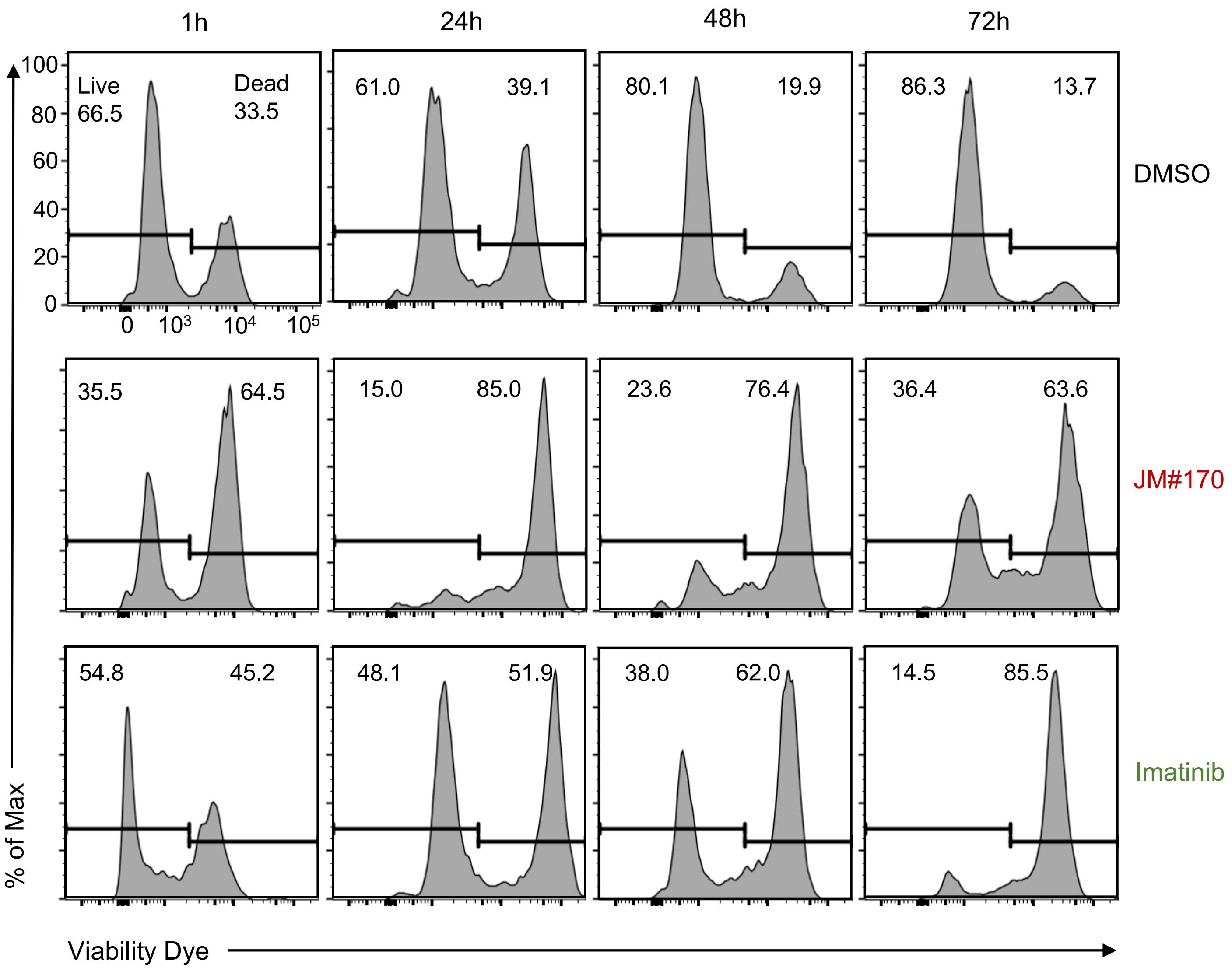

FACS analysis for live-dead staining of JM#170 treated BCR-ABL1 cells. Representative histograms (n=3) showing live-dead gating for BCR-ABL1 cells treated with DMSO, 10µM of JM#170 and 1µM of Imatinib for the indicated time period and stained with viability dye. The gating remains same for all measurements performed on a particular time point. However, slight adjustment of gating may be necessary from one time point to the other.

Supplementary Figure S3

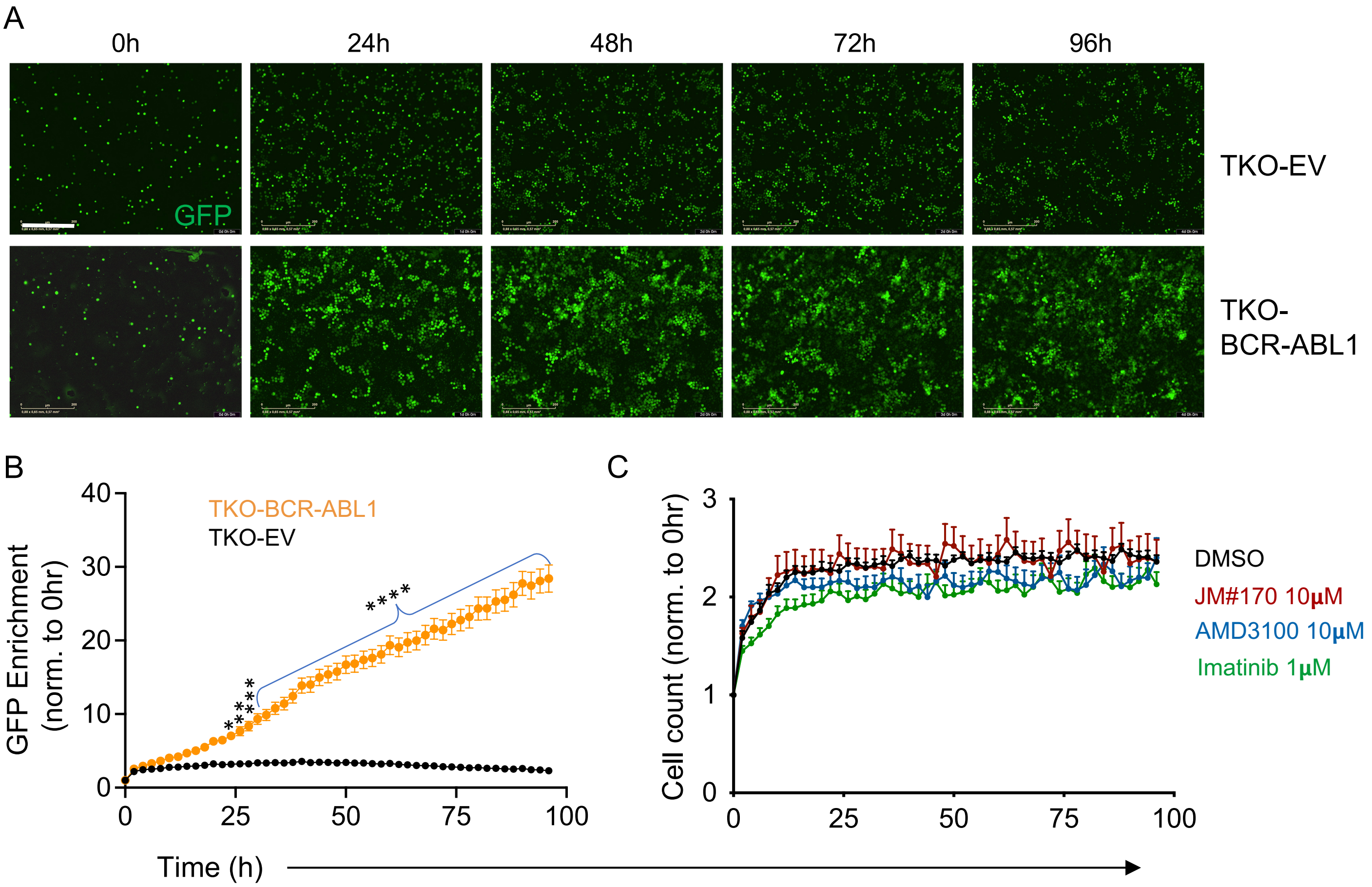

JM#170 does not affect the growth of WT BM derived B cells *in vitro*. A. Real-time imaging of GFP+ cell enrichment of TKO cells transduced either with empty pMIG vector (TKO-EV) or BCR-ABL1 construct (TKO-BCR-ABL1) for 96 hours. Scale bar- 200µm. B. Quantification of GFP+ cell enrichment in TKO-EV and TKO-BCR-ABL1 cells as depicted in A. The GFP+ cell count is normalized with respect to the count at 0 hour. Graph represents mean ± SEM, One representative experiment of n=3 is shown. Statistical analysis- two-way ANOVA with Dunnett's multiple comparison test. *$p<0.05$, **$p<0.01$, ***$p<0.001$, ****$p<0.0001$. C. Quantification of cell number in DMSO (black), 10µM JM#170 (dark red), 10µM AMD3100 (blue) and 1µM Imatinib (green) treated WT bone marrow B cells over 96 hours. The cell count for each time point for each treatment is normalized with respect to the corresponding count at 0 hour. Graph represents mean ± SEM, One representative experiment of n=2 is shown. Statistical analysis- two-way ANOVA with Dunnett's multiple comparison test.

Supplementary Figure S4

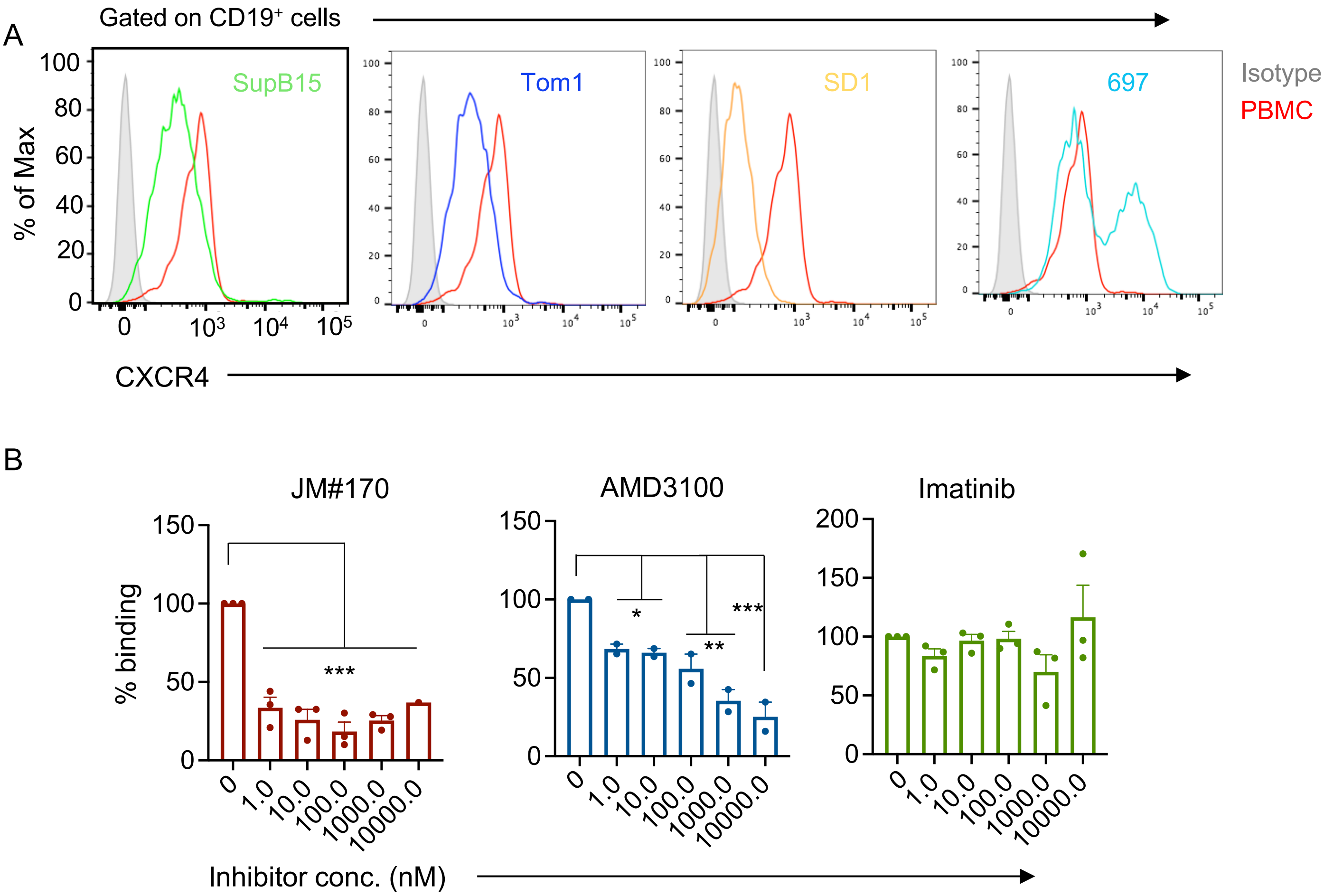

JM#170 and AMD3100 inhibit CXCR4 in human ALL cell lines. A. Histogram showing flow cytometric analysis of CXCR4 expression on the surface of the indicated human ALL cell lines in comparison to healthy human blood derived B cells (red line). Grey filled histogram represents isotype control. B. Anti-CXCR4 antibody (clone# 12G5) competition assay in SupB15 cells in presence of indicated concentrations of JM#170 (left panel), AMD3100 (middle panel) and Imatinib (right panel). Bar represents mean ± SEM, n=2-3. Statistical analysis- one-way ANOVA with Dunnett's multiple comparison test. *p<0.05, **p<0.01, ***p<0.001.

Supplementary Figure S5

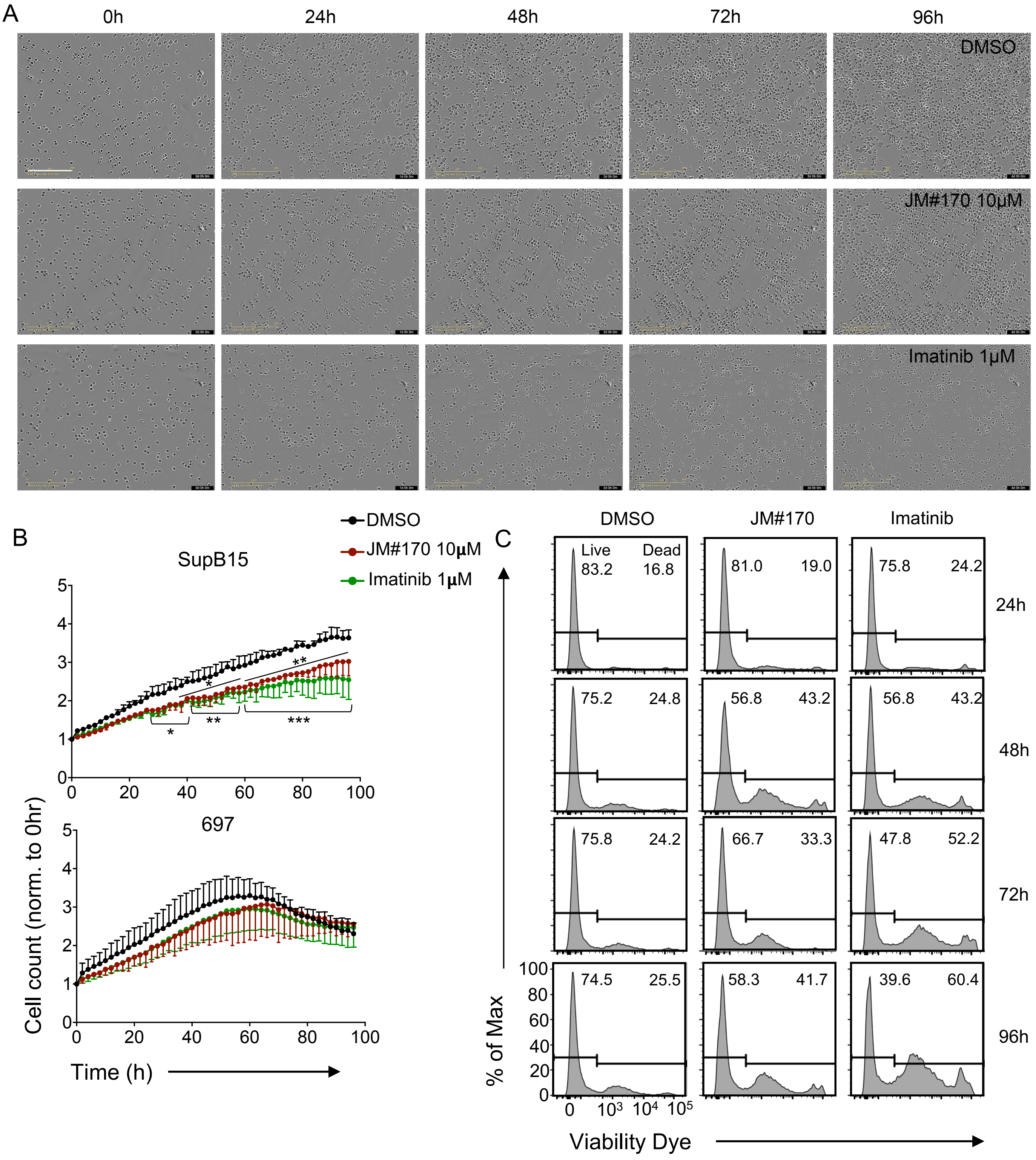

Effect of JM#170 and Imatinib on human ALL cell lines. A. Real-time imaging of SupB15 cells treated with DMSO, 10µM JM#170 or 1µM Imatinib over 96 hours. Phase contrast images are representative of one experiment out of n=3. Scale bar-200µm. B. Quantification of cell number in DMSO (black), 10µM JM#170 (dark red) and 1µM Imatinib (green) treated SupB15 (upper panel) and 697 (lower panel) cells over 96 hours. The cell count for each time point for each treatment is normalized with respect to the corresponding count at 0 hour. Graph represents mean ± SEM, n=3. Statistical analysis- two-way ANOVA with Dunnett's multiple comparison test. *p<0.05, **p<0.01, ***p<0.001. C. Representative histograms (n=3) showing live-dead gating for SupB15 cells treated with DMSO, 10µM of JM#170 and 1µM of Imatinib for the indicated time period and stained with viability dye.

Supplementary Figure S6

A

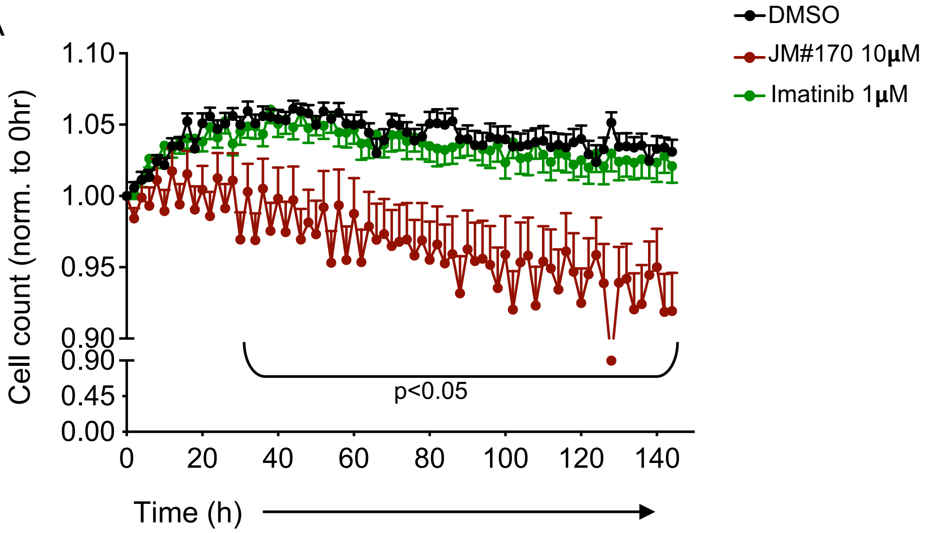

B

Time (h)	DMSO		JM#170 10µM			Imatinib 1µM		
	Mean	SEM	Mean	SEM	p (wrt DMSO)	Mean	SEM	p (wrt DMSO)
0	1	0	1	0	ns	1	0	ns
24	1.048	0.006	1.013	0.018	ns	1.041	0.008	ns
48	1.058	0.006	0.982	0.023	***	1.048	0.011	ns
72	1.05	0.007	0.968	0.027	***	1.043	0.011	ns
96	1.042	0.01	0.952	0.028	***	1.032	0.013	ns
120	1.04	0.01	0.925	0.025	***	1.026	0.012	ns
144	1.031	0.009	0.92	0.027	***	1.021	0.012	ns

Effect of JM#170 and Imatinib on primary Ph+ALL xenograft cells. A. Real-time imaging and quantification of cell number of xenograft cells treated with DMSO (black), 10µM JM#170 (dark red) and 1µM Imatinib (green) over 120 hours. The absolute cell count for each time point for each treatment is normalized with respect to the corresponding count at 0 hour. Graph represents mean ± SEM, n=2. Statistical analysis- two-way ANOVA with Dunnett's multiple comparison test. ***$p<0.001$. B. Table representing the normalized values for quantification of the above graph for specified time points with corresponding statistical significance. wrt: with respect to.

Supplementary Figure S7

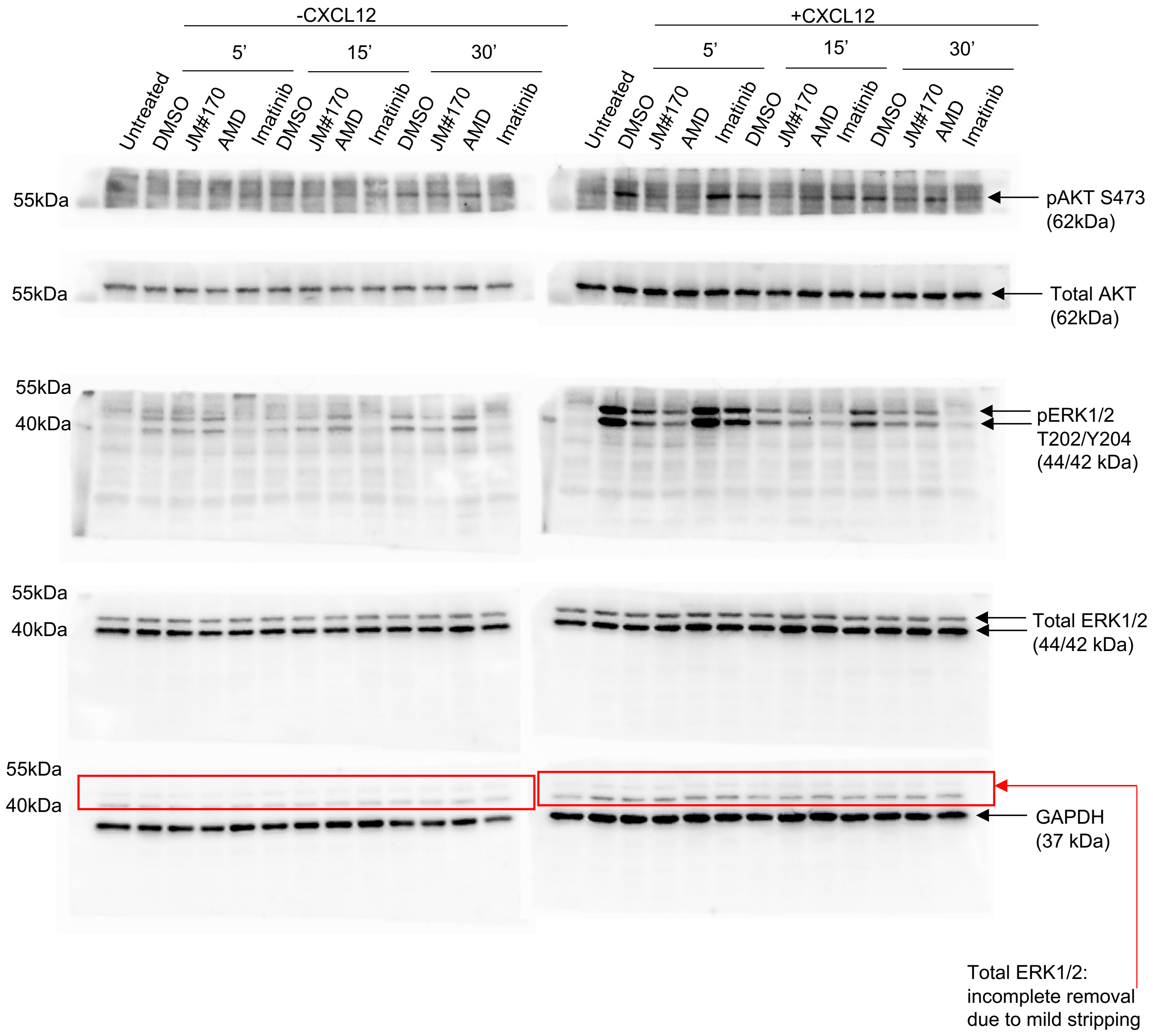

Images of the full membranes of the western blot used in Figure 3A: Uncropped blots are represented for the indicated antibodies along with the molecular weight markers.

Supplementary Figure S8

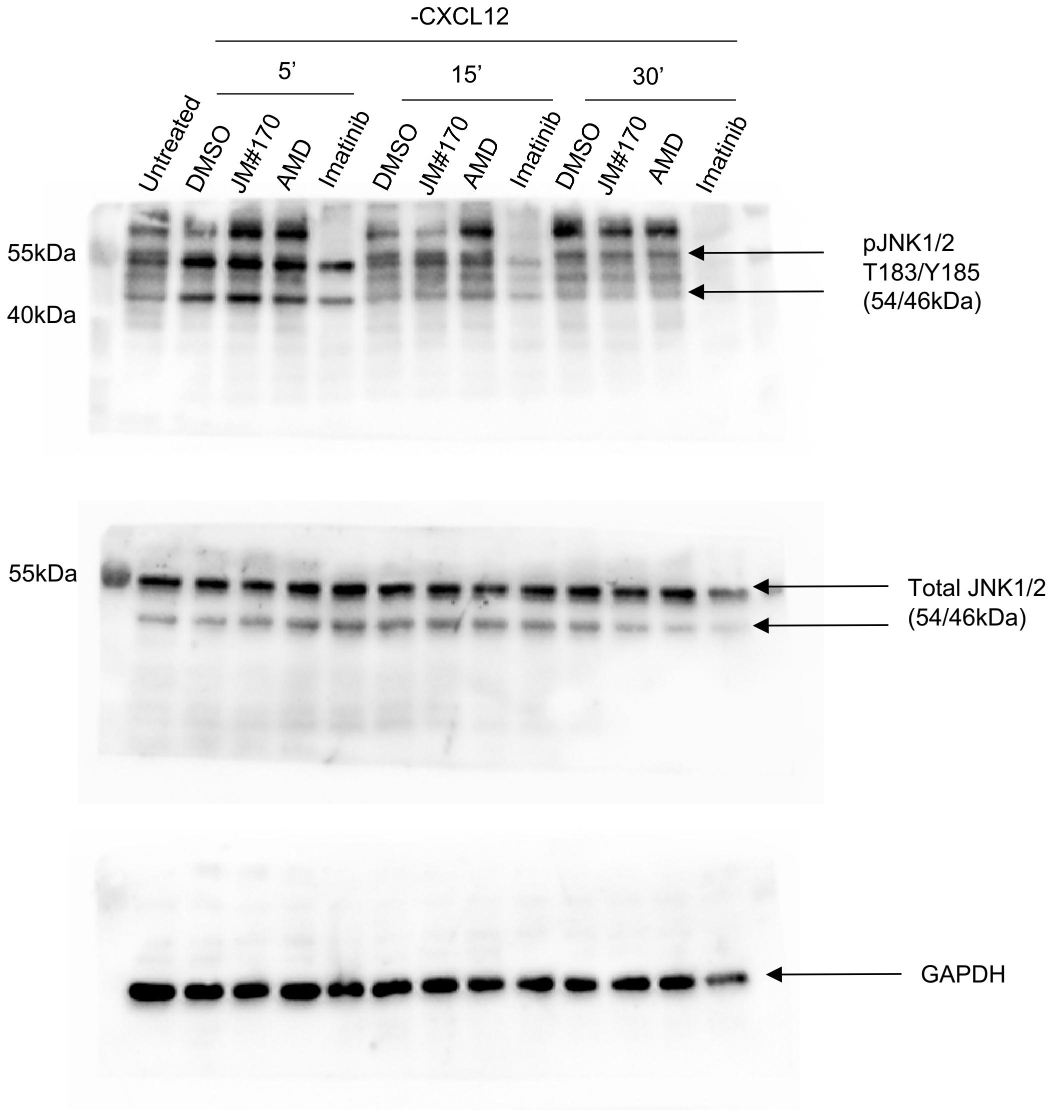

Images of the full membranes of the western blot used in Figure 4A: Uncropped blots are represented for the indicated antibodies along with the molecular weight markers.

Supplementary Table S1: Quantification of GFP^+ BCR-ABL cells upon treatment with JM#170 and Imatinib either alone or in combination.

Time (h)	DMSO		JM#170 10mM		Imatinib 100nM		Imatinib 100nM + JM#170 10mM	
	Mean	SEM	Mean	SEM	Mean	SEM	Mean	SEM
0	1	0	1	0	1	0	1	0
2	1.07	0.017	1.182	0.092	1.044	0.01	0.938	0.069
4	1.727	0.582	1.158	0.103	1.088	0.007	0.87	0.133
6	1.787	0.55	1.153	0.119	1.121	0.03	0.834	0.17
8	1.835	0.472	1.177	0.133	1.205	0.047	0.839	0.208
10	1.567	0.099	1.211	0.15	1.25	0.079	0.833	0.232
12	2.225	0.6	1.236	0.166	1.356	0.108	0.858	0.27
14	2.19	0.431	1.263	0.183	1.416	0.129	0.87	0.299
16	2.3	0.386	1.304	0.193	1.509	0.163	0.887	0.323
18	2.458	0.405	1.346	0.212	1.591	0.196	0.899	0.346
20	2.58	0.37	1.373	0.221	1.705	0.243	0.91	0.368
22	2.839	0.478	1.408	0.235	1.78	0.283	0.914	0.387
24	2.925	0.357	1.443	0.252	1.892	0.32	0.911	0.4
26	3.201	0.47	1.485	0.272	1.989	0.377	0.905	0.402
28	2.901	0.039	1.523	0.29	2.105	0.42	0.913	0.41
30	3.291	0.185	1.579	0.31	2.216	0.467	0.912	0.414
32	3.542	0.197	1.63	0.331	2.358	0.509	0.911	0.423
34	3.27	0.219	1.701	0.354	2.45	0.566	0.884	0.413
36	3.945	0.195	1.757	0.379	2.628	0.606	0.88	0.414
38	4.094	0.151	1.832	0.41	2.705	0.647	0.862	0.402
40	4.205	0.016	1.889	0.43	2.865	0.679	0.866	0.403
42	4.761	0.349	1.973	0.47	2.978	0.729	0.851	0.399
44	5.025	0.352	2.052	0.495	3.127	0.825	0.847	0.4
46	5.269	0.394	2.121	0.524	3.257	0.868	0.838	0.4
48	5.257	0.069	2.185	0.557	3.421	0.936	0.843	0.404
50	5.549	0.107	2.253	0.584	3.587	1.015	0.84	0.402
52	5.662	0.127	2.339	0.615	3.797	1.082	0.846	0.404
54	6.226	0.174	2.41	0.639	3.945	1.149	0.84	0.394
56	5.414	1.05	2.485	0.664	4.201	1.251	0.855	0.41
58	6.589	0.097	2.575	0.711	4.351	1.311	0.849	0.407
60	7.364	0.236	2.662	0.741	4.647	1.447	0.867	0.417
62	7.329	0.122	2.744	0.779	4.834	1.493	0.866	0.418
64	7.617	0.242	2.808	0.799	5.109	1.564	0.893	0.427
66	7.111	1.115	2.914	0.841	5.333	1.668	0.906	0.433
68	7.261	1.377	3.006	0.87	5.636	1.786	0.93	0.452
70	9.37	0.379	3.152	0.92	5.937	1.869	0.946	0.463
72	9.396	0.088	3.222	0.938	6.244	2.005	0.979	0.485
74	9.856	0.024	3.33	0.971	6.509	2.053	1.001	0.497
76	10.638	0.291	3.422	1.001	6.904	2.146	1.04	0.517
78	9.612	1.113	3.545	1.041	7.196	2.238	1.069	0.529
80	10.587	0.69	3.674	1.07	7.625	2.365	1.131	0.571
82	9.863	1.682	3.798	1.1	7.888	2.389	1.157	0.591
84	11.832	0.373	3.917	1.125	8.377	2.503	1.2	0.614
86	11.151	1.378	4.053	1.165	8.677	2.543	1.234	0.634
88	12.317	0.67	4.173	1.2	9.028	2.479	1.299	0.667
90	14.002	0.774	4.316	1.25	9.301	2.511	1.333	0.692
92	14.26	0.515	4.391	1.25	9.858	2.651	1.406	0.741
94	11.758	2.328	4.545	1.298	10.168	2.605	1.444	0.769
96	15.849	1.413	4.679	1.336	10.605	2.625	1.517	0.82

The absolute cell numbers for each treatment are normalized with respect to the corresponding cell number at 0h.
